# Supplementary material for: Prospective Evaluation of Local Sustained Release of Celecoxib in Dogs with Low Back Pain
Source: Pharmaceutics. 2021 Jul 30;13(8):1178. doi: 10.3390/pharmaceutics13081178 (PMC8398998; doi:10.3390/pharmaceutics13081178)
Supplement: Supplementary file 1 [file pharmaceutics-13-01178-s001.zip › pharmaceutics-1284129-supplementary.pdf]

pharmaceutics

MDPI

Prospective Evaluation of Local Sustained Release of Celecoxib in Dogs with Low Back Pain

Tijn Wiersema, Anna R. Tellegen, Martijn Beukers, Marijn van Stralen, Erik Wouters, Mandy van de Vooren, Nina Woike, George Mihov, Jens C. Thies, Laura B. Creemers, Marianna A. Tryfonidou and Björn P. Meij

Table S1. Overview of dogs with degenerative lumbosacral stenosis included in the study.

Dog #	Age (years) at time of	Sex	Weight (Kg)	Breed	Purpose	Treatment	Pfirrmann Grade	Disc protrusion %	Telescoping/Ligamentum Flavum	Modic changes	Spinal Nerve Swelling	Intervertral Foramen Stenosis	Facet Joint Arthrosis
1	4,7	FN	51,7	Rhodesian Ridgeback	Companion	Placebo	3	25%-50%	Mild	3	No	Subtle L and R	Mild
2	1.2	M	31.0	Labrador Retriever	Service	Placebo	2	<25%	Mild	0	No	No	No
3	1.9	FN	32.5	Old English Bulldog	Companion	CXB-PEAM	3	<25%	Mild	1 and 3	Yes	Mild L and R	Mild
4	6.6	MN	14.8	Beagle	Companion	Placebo	3	25%-50%	No (very subtle)	0	No	No	No
5	10.3	M	38.9	Labrador Retriever	Service	CXB-PEAM	4	<25%	Mild	3	No	No	No
6	3.8	M	18.5	Border Collie	Sport	Placebo	3	25%-50%	Mild	0	No	Mild L and R	No
7	3.4	FN	28.0	Labrador Retriever	Companion	CXB-PEAM	2	<25%	Mild	0	No	No	No
8	3.3	MN	25.0	Labrador Retriever	Service	Placebo	2	25%-50%	No	0	Yes	Marked right	Mild
9	3.7	MN	28.0	Labrador Retriever	Service	CXB-PEAM	2	<25%	No	0	No	No	Mild
10	6.0	FN	23.4	Labradoodle	Companion	CXB-PEAM	4	<25%	No (very subtle)	3	Yes	Moderate L and R	No
11	1.7	MN	21.4	Labradoodle	Companion	CXB-PEAM	2	<25%	No	3	No	No	No
12	6.2	FN	28.3	German Shepherd	Companion	CXB-PEAM	3	>50%	No	0	No	Moderate L and R	Subtle
13	2.8	FN	12.6	Mixed breed	Companion	CXB-PEAM	2	<25%	No	3	No	No	No
14	7.9	F	27.0	Golden Retriever Mix	Service	Placebo	3	25%-50%	Mild	0	No	No	No
15	5.3	FN	27.0	Weimaraner	Companion	CXB-PEAM	2	25%-50%	Mild	0	No	Subtle L and R	Subtle
16	1.9	FN	40.4	Rottweiler	Companion	CXB-PEAM	4	<25%	No	1 and 3	Yes	Moderate L and R	No
17	1.8	MN	32.6	Labrador Retriever	Companion	Placebo	2	0%	No	0	No	No	No
18	7.1	FN	25.8	Flatcoat Retriever	Companion	CXB-PEAM	4	25%-50%	No	0	No	No	No
19	6.1	MN	33.7	German Shepherd	Companion	CXB-PEAM	4	25%-50%	Mild	0	Yes	Mild/moderate L >	Mild
20	2.1	MN	32.4	Golden Retriever Mix	Companion	CXB-PEAM	2	<25%	No	0	No	No	No
21	9.6	MN	24.6	Spanish Water Dog	Companion	CXB-PEAM	4	25%-50%	Mild	3	No	Mild/moderate R>	No
22	5.0	MN	22.9	Small Münsterlander	Companion	CXB-PEAM	2	<25%	No (very subtle)	0	No	No	No
23	1.4	M	30.6	Labrador Retriever	Service	Placebo	2	<25%	No	0	No	No	No
24	2.9	M	30.2	Labrador Retriever	Service	CXB-PEAM	2	<25%	No (very subtle)	0	No	No	No
25	6.2	FN	13.2	English Cocker	Sport	CXB-PEAM	3	<25%	No	0	No	No	No
26	4.9	MN	27.5	Basset Hound	Companion	Placebo	3	<25%	No	0	Yes	Moderate left	No
27	4.3	FN	29.6	Golden Retriever Mix	Service	CXB-PEAM	2	<25%	No	0	No	No	No
28	7.2	M	32.5	Labrador Retriever	Service	Placebo	3	<25%	No (very subtle)	0	No	No	No
29	7.6	MN	29.7	Golden Doodle	Service	CXB-PEAM	4	25%-50%	No	1	No	Moderate left	No
30	6,2	M	30,1	Labrador Retriever	Service	CXB-PEAM	2	<25%	No	0	No	No	No

F: Female, FN: Female Neutered, M: Male, MN: Male Neutered, CXB-PEAM: Celecoxib loaded polyesteramide microspheres, L: Left, R: Right

Table S2. Magnetic Resonance Imaging findings for male and female dogs with degenerative lumbosacral stenosis at baseline.

MRI	Male (n = 18)	Female (n = 12)	P(*)
Pfirrmann Grade			
2	10	4	0.505
3	4	5	
4	4	3	
Disc Protrusion ≥25% (n,%)	6 (33%)	5 (42%)	0.712
Modic Changes Present (n,%)	4 (22%)	5 (42%)	
**T2 relaxation time**			
L6-L7 (mean, sd)	169 (85)	152 (56)	0.421
L7-S1 (mean, sd)	132 (73)	117 (78)	0.339

(*) Statistical significance based on Fisher's exact tests for categorical data (Pfirrmann grade, disc protrusion, Modic changes) and based on Mann–Whitney test for continuous data (T2 relaxation times).

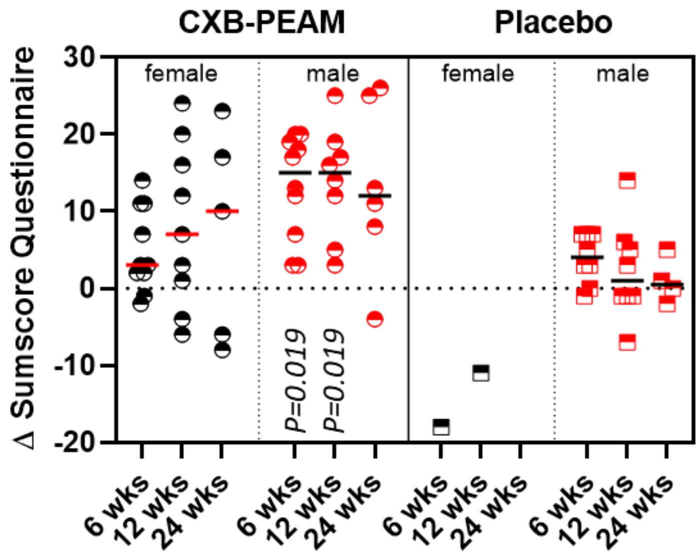

Figure S1. Subgroup scatterplots demonstrating the change in time of the questionnaire sumscore (Δsumscore) (median indicated) in the subgroups (male/female) of 30 dogs suffering from degenerative lumbosacral stenosis treated with intradiscal injection of celecoxib-polyesteramide microspheres (PEAMs) or unloaded PEAMs (placebo). After 12 weeks follow up the label was opened; the data of the 24 week follow up was not subjected to statistical analysis.
